# Predicting the target specialty of referral notes to estimate per-specialty wait times with machine learning

**DOI:** 10.1371/journal.pone.0267964

**Published:** 2022-05-12

**Authors:** Mohamed Abdalla, Hong Lu, Bogdan Pinzaru, Frank Rudzicz, Liisa Jaakkimainen

**Affiliations:** 1 Department of Computer Science, University of Toronto, Toronto, Canada; 2 Vector Institute for Artificial Intelligence, Toronto, Canada; 3 ICES, Toronto, Canada; 4 Unity Health Toronto, Toronto, Canada; 5 Department of Family and Community Medicine, University of Toronto, Toronto, Canada; 6 Department of Family and Community Medicine, Sunnybrook Health Sciences Centre, Toronto, Canada; Northeastern University, UNITED STATES

## Abstract

**Background:**

Currently, in Canada, existing health administrative data and hospital-inputted portal systems are used to measure the wait times to receiving a procedure or therapy after a specialist visit. However, due to missing and inconsistent labelling, estimating the wait time prior to seeing a specialist physician requires costly manual coding to label primary care referral notes.

**Methods:**

In this work, we represent the notes using word-count vectors and develop a logistic regression machine learning model to automatically label the target specialist physician from a primary care referral note. These labels are not available in the administrative system. We also study the effects of note length (measured in number of tokens) and dataset size (measured in number of notes per target specialty) on model performance to help other researchers determine if such an approach may be feasible for them. We then calculate the wait time by linking the specialist type from a primary care referral to a full consultation visit held in Ontario, Canada health administrative data.

**Results:**

For many target specialties, we can reliably (*F*_1_Score ≥ 0.70) predict the target specialist type. Doing so enables the automated measurement of wait time from family physician referral to specialist physician visit. Of the six specialties with wait times estimated using both 2008 and 2015 data, two had a substantial increase (defined as a change such that the original value lay outside the 95% confidence interval) in both median and 75th percentile wait times, one had a substantial decrease in both median and 75th percentile wait times, and three has non-substantial increases.

**Conclusions:**

Automating these wait time measurements, which had previously been too time consuming and costly to evaluate at a population level, can be useful for health policy researchers studying the effects of policy decisions on patient access to care.

## Introduction

Reliable information about the time spent waiting for health care services is a critical metric for measuring health system performance. Currently, in Canada, existing health administrative data or hospital-inputted portal systems are used to measure the wait times from a specialist physician visit to receiving a procedure or therapy and these measures are regularly displayed on platforms accessible to the public, health care planners, and providers [1, 2].

However, patients experience waits prior to seeing a specialist physician and, indeed, surveys conducted in several jurisdictions demonstrate that Canadians experience long wait times in this regard. In 2017, among eleven developed countries surveyed, Canada ranked 10^th^ on access and 11^th^ on timeliness of health care [3]. The 2019 Fraser Institute survey of Canadian provinces found wait times from family physician (FP) referral to seeing a specialist was 8 weeks which represented an increase from the previous survey [4]. While these surveys provide needed information about wait times, surveys are expensive, time consuming, and the data are subject to recall bias. Alternatively, electronic medical records (EMRs) can be used to arrive at wait time estimates. However, due to poor and missing labels, target specialty physician labelling is currently a task requiring manual human labelling, something we wish to automate to increase the number of referrals labelled and decrease the cost and time associated with conducting such studies.

Over 86% of Canadian FPs now use EMRs for routine clinic care [5]. Alongside is the increased experience of transforming the information held within FP EMRs into data for research and quality improvement initiatives [6–10]. However, using FP EMR data to facilitate real-world and real-time wait times estimation is challenged by its unstructured and free-text format, and lack of trust-worthy labels. Previously, we demonstrated the feasibility of using FP EMR data, linked to health administrative date, to measure specialist wait times [11]. However, this method required abstractors to codify the unstructured EMR data, which limits our ability to provide these data in a timely manner. In this work, we sought to develop a machine learning model to automate the task of these human abstractors: labelling the target specialty of a referral note.

### Related works

In this subsection, we will explore related work. We categorize related work into two categories: 1) work measuring wait-times in healthcare system settings, and 2) work performing text classification on referral notes or other types of clinical notes.

Traditionally, work measuring wait-times in healthcare systems do not engage in machine learning prediction to enable the measurement. This is because either the required labels can be found within the administrative data [12, 13], or because they have used manual effort to label the notes [11].

There is work which develops machine learning applications that reduce wait-times [14], or attempt to predict length-of-stay [15] (which is related to patient wait-times, though in a referral setting–the focus of our work). Gonçalves *et al*. [16] attempted to predict patient wait-times between triage to observation. Lin et al. [17] attempt to predict wait-times for patients for Pediatric Ophthalmology outpatient clinic using patient characteristics and other relevant features. Outside of the medical domain (e.g., business or government offices) other work has built models to predict expected wait-times given historical data [18, 19].

We have been unable to find other works which used machine learning on free text clinical notes to label the target specialty of referral notes to enable wait-time estimation–most machine learning work attempts to predict the wait-time directly. Bauder *et al*. [20] attempted to classify medical provider specialties from their billed codes (a different mode of input) to detect fraud (a different goal).

There is a large body of work applying machine learning to clinical notes to perform classification. Amongst the many classification tasks are identification of patients with pathologies [21], prediction of admission [22], and predicting high-cost users [23] among many other tasks (e.g., medical subdomain classification [24]). To perform the classification these works train and evaluate a variety of machine learning models such as: Logistic Regression [21–23], Random Forests [21, 22], Gradient-boosted models [22], Support Vector Machines [25], Shallow Neural Networks [21], and deep neural networks [22]. The results of which model performs better is heavily dependent on the dataset and task at hand.

## Methods

**Fig 1:**
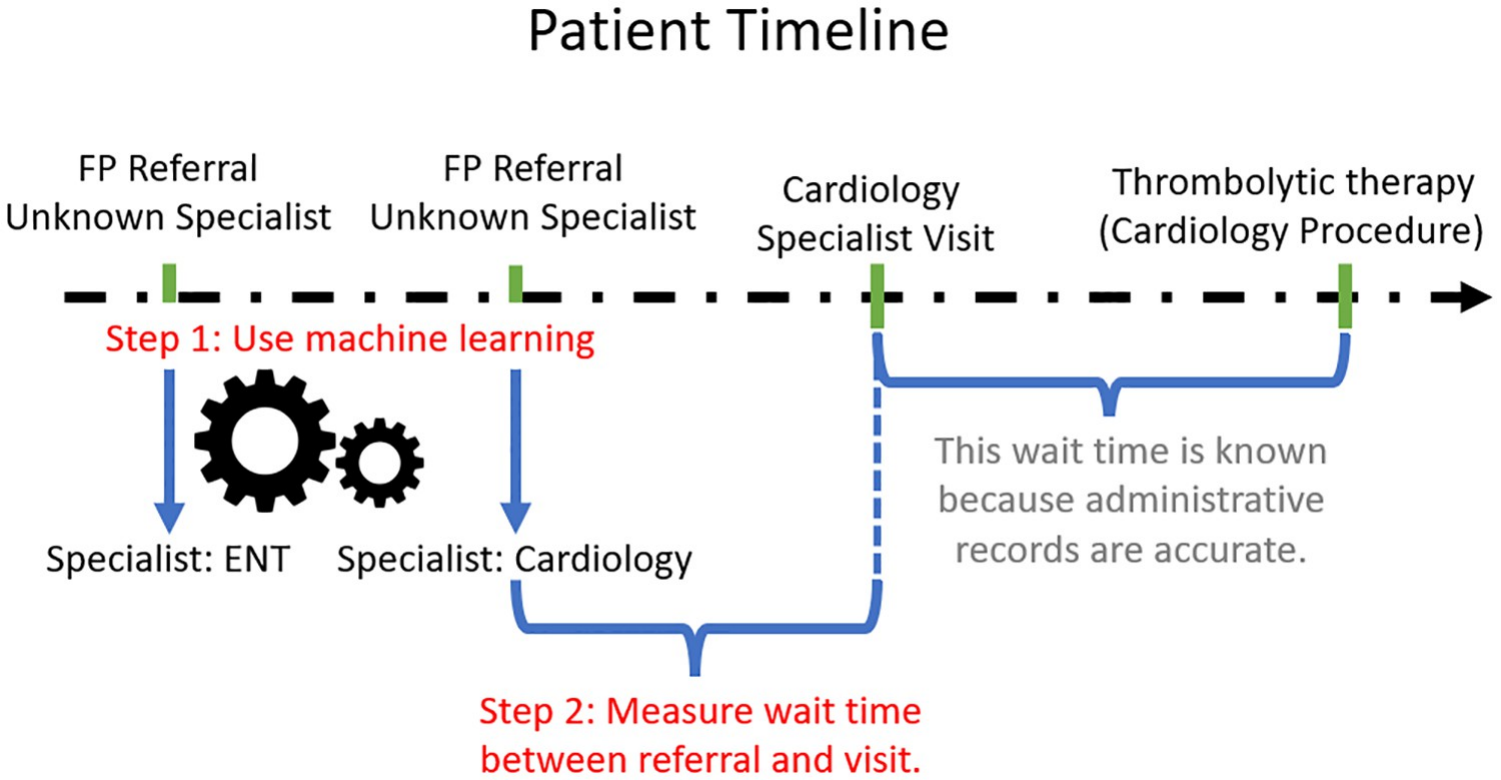
A simplified visualization of a patient’s healthcare timeline. The time between specialist visit and procedure is known because administrative records are accurate. We show our methodology in two steps: 1) Using machine learning to predict the target specialty of referral notes, and 2) Measuring the wait time between referral and consultation visit of pair specialties.

In this study, we sought to develop an automated method to identify the target specialist physician type from referral notes using machine learning (ML), Step 1 in Fig 1. We test the performance of our trained models on various target specialties and explore how the characteristics of the referral notes affect classification performance. For the specialties with acceptable performance (defined as a precision of at least 0.70 and a sensitivity of at least 0.30), we present the calculated wait times from FP referral to target specialist visit, Step 2 in Fig 1. We also compare estimated wait times against the manually curated results from 2008 [11], and the wait times produced by changing the confidence threshold.

### Dataset

#### Referral notes and administrative data

We use FP EMR data linked to Ontario health administrative data held at ICES called EMRPC. ICES is an independent non-profit research institute and has legal status under Ontario’s health information privacy law that allows it to collect and analyze health care and demographic data, without consent, for health system evaluation and improvement [26]. Projects that use data collected by ICES under section 45 of PHIPA, and use no other data, are exempt from REB review. The use of the data in this project is authorized under section 45 and approved by ICES’ Privacy and Legal Office. EMRPC collects EMR data from FPs distributed across Ontario (currently just over 350,000 rostered patients from 403 FPs in 46 geographically distinct clinics) [27, 28]. EMRPC extracts the entire EMR of FP patients, including their cumulative patient profile (CPP), progress notes, consultation notes, radiological and laboratory tests, hospital and emergency discharge notes, and referrals. We used free text in referral notes to predict target specialty physician and used physician claims in the Ontario Health Insurance Plan (OHIP) database to determine when the specialist visits happened. These datasets were linked using unique encoded identifiers and analyzed at ICES.

Our study cohort includes patients with a valid health card number and date of birth who had at least one FP referral made in 2015 (N = 56,285). Patients are included if their family physician had signed a data sharing agreement with ICES, had an active practice, and were completely utilizing the EMR in their practice.

We use the free text of the referral notes as input to our classification model. However, as the labels associated with the referral notes in the EMR are incomplete and untrustworthy, we had to generate the “gold standard” labels (i.e., the target specialist for each referral note). To do this, rather than relying on manual annotation of a random subset of referral notes (a costly endeavor), we use the OHIP full consultation claims (i.e., billing data) to generate gold-standard labels. To ensure that we correctly paired referral notes with their correct target specialist for our gold labels, we only consider patients who have only one referral note and one specialist visit in 2015 (N≈16,840). This is because considering patients with more than a single referral or a single specialist visit causes issues because of the possible combinations of assignments (e.g., a patient with three referrals and three different specialist visits can have up to 6 different pairings between each referral note and specialist visit). Having only one referral and one specialist visit makes it increasingly likely that the referral was to the visited specialist. Table 1 presents the number of notes in the training and testing set for each specialist type.

**Table 1:** The number of notes per specialist type. Notes with fewer than 6 tokens were not counted as they were not informative, per manual checking. The specialty “internal” was also removed as the types of medical consultation done by an internal specialist overlaps with many individual specific specialties such as cardiology, gastroenterology, among others. If present, the names within parentheses represent how the specialty is represented in later figures.

Specialist Type	Number of Notes
General Surgery	2351
Gastroenterology	1714
Cardiology	489
Obstetrics and Gynecology (OBGYN)	1958
Plastic Surgery (Plastic)	980
Otolaryngology (ENT)	1931
Dermatology	3014
Neurology	509
Urology	948
Paediatrics	148
Rheumatology	296
Ophthalmology	475
Physical medicine	156
Haematology	92
Genetics	15
Endocrinology	219
Family Medicine	137
Immunology	236
Psychiatry	241
Nephrology	65
Geriatrics	32
Respiratory	220
Infectious disease	24
Anesthesia	<5*

*ICES privacy guidelines require us to suppress small values.

### Machine-learning pipeline for classification

The machine learning pipeline introduced in this section is developed and evaluated on the subset of data described above using the Python package Scikit-learn [29]. The machine learning pipeline follows these steps:

1. Preprocessing.
2. Vectorization
3. Model training and selection.

#### Preprocessing

For preprocessing the referral notes, we convert all the text to lowercase and remove all punctuation and numbers. Manual examination of notes with fewer than 6 tokens in total resulted in the observation that most of these notes were note informative; most of these notes were not true referral notes or just included a de-identified name and a date (i.e., information which is not useful for classification). As such, we removed notes with fewer than 6 tokens in our dataset. The fact that these notes are not informative for prediction is supported by our experiment on the effect of length of note on performance below. We experiment with stemming [30] and not-stemming each token in the referral notes using the Snowball stemmer implemented in the Python package NLTK [31].

#### Vectorization

To convert the free-text of clinical notes into numeric vectors amenable to machine learning, we experiment between word count vectors and term-frequency inverse document frequency (tf-idf) vectors [32]. Word count vectors represent a document using a dense numeric vector where each number in the vector is the count of the number of occurrences of a given word type (one per index) in the document. We consider all individual tokens (i.e., ‘unigrams’) that occurred at least 5 times. The reason for considering all tokens is that we did not want to engage in the act of creating a specialized vocabulary per specialty (this approach is supposed to provide an alternative to costly manually labelling). The reason behind excluding tokens that did not occur at least 5 times across all notes is because such tokens tend to be rare-misspellings and would be useful in helping the model generalize. This is also standard operating procedure in text classification. Tf-idf vectors are similar to count vectors in that each index in the vector refers to an individual word type; however, rather than simply representing the number of occurrences, this approach adjusts how often each word occurs across all *other* notes to adjust for words that occur frequently in all documents (e.g., functional words such as *‘the*’*)*. For each preprocessing type (i.e., stemming and not stemming), we experiment with both types of vectorization.

#### Model training

We experiment with two different classification algorithms: lasso and ridge logistic regression. In the Related Works section, we demonstrate that there are a wide variety of models that can be used to perform such classification. We selected these two variants of logistic regression for multiple reasons: 1) previous literature has shown logistic regression out-performs other classifiers on datasets of similar size [33–35], 2) it is a simple, yet effective, method that can easily be replicated by other sites who may not have the required computational power to apply more demanding approaches, 3) logistic regression models tend to be well calibrated (without external modification unlike deeper neural networks [36]) thus enabling our later analysis, 4) the weights used by logistic regression models are interpretable and can be used for further analysis [37]. For both, we experiment with different hyperparameters (e.g., amount of regularization). The complete set of possible training combinations attempted in this task is shown in Table 2. For each of the combinations listed in Table 2, we use 5-fold cross-validation to measure performance. Each trained classifier was trained as a binary classifier learning to predict only a single specialty (i.e., one-vs-rest).

**Table 2:** All possible options for each step of the machine learning pipeline. All possible combinations of these options were trained for each target specialty (for a total of 80 models per specialty).

Preprocessing	Vectorization	Model Type	Regularization	Class Weight
No stemming	Count vectors	Ridge Regression	0.001	‘None’
Stemming	Tf-idf vectors	Lasso Regression	0.01	‘Balanced’
			0.1	
			1	
			10	

## Results

For each target specialty presented in Table 1, we train multiple models with various settings and hyper-parameters; all possible combinations are presented in Table 2. Depending on the target application, different performance metric optimizations may be desired (e.g., high recall at the cost of precision) [38, 39]. To provide a holistic overview of model performance, we select and present the best model performance based on three optimization schemes:

1. Choosing the model with the highest precision/positive predictive value (PPV) while ensuring a recall (alternatively: sensitivity) of at least 0.3. When no set of hyperparameters result in a model with a recall of at least 0.3, the model with highest precision is presented.
2. Choosing the model with the highest recall while ensuring a precision of at least 0.3. When no set of hyperparameters result in a model with a minimum precision of at least 0.3, the model with the highest recall is presented.
3. Choosing the model with the highest *F*_1_Score.

We ensure a lower limit on the recall or precision for the first two optimization schemes because for most specialties there are a set of hyperparameters which result in near perfect performance of one metric at the extreme cost of the other (e.g., PPV: 0.97, Sensitivity: 0.01). However, these models are not likely to be useful in the clinical setting. We evaluate the performance of the models using 5-fold cross-validation and present the results in Table 3.

**Table 3:** Precision, recall, and *F*_1_Score for each optimization for each specialty found in Table 1. We do not present evaluate any models for the “aesthesia” class as there were fewer examples than folds. Specialties which are bolded pass our performance threshold.

Specialist Type	Models with Recall at least 0.3			Models with Precision at least 0.3			Models with Highest *F*_1_Score		
	PPV	SEN	*F*_1_	PPV	SEN	*F*_1_	PPV	SEN	*F*_1_
**General surgery**	**0.72**	**0.31**	**0.42**	0.49	0.73	0.58	0.49	0.73	0.58
Gastroenterology	0.66	0.42	0.51	0.42	0.81	0.55	0.47	0.80	0.59
**Cardiology**	**0.83**	**0.41**	**0.55**	0.31	0.80	0.43	0.72	0.53	0.61
**Obstetrics/Gynecology**	**0.95**	**0.49**	**0.64**	0.72	0.83	0.77	0.76	0.82	0.79
Plastic Surgery	0.57	0.41	0.47	0.39	0.71	0.49	0.46	0.59	0.51
**ENT**	**0.98**	**0.42**	**0.56**	0.68	0.82	0.74	0.85	0.72	0.78
**Dermatology**	**0.85**	**0.51**	**0.62**	0.66	0.83	0.74	0.66	0.83	0.74
**Neurology**	**0.82**	**0.33**	**0.46**	0.50	0.75	0.60	0.55	0.68	0.61
**Urology**	**0.91**	**0.52**	**0.65**	0.49	0.82	0.61	0.79	0.65	0.71
Paediatrics	0.38	0.38	0.36	0.33	0.54	0.38	0.33	0.54	0.38
**Rheumatology**	**0.78**	**0.38**	**0.51**	0.35	0.61	0.44	0.78	0.38	0.51
Ophthalmology	0.51	0.41	0.39	0.31	0.64	0.37	0.37	0.57	0.39
Physical medicine	0.25	0.33	0.28	0.32	0.13	0.18	0.23	0.53	0.32
Haematology	0.25	0.33	0.21	0.36	0.21	0.22	0.25	0.33	0.21
Genetics	0.32	0.4	0.25	0.32	0.4	0.25	0.80	0.27	0.40
Endocrinology	0.62	0.31	0.41	0.31	0.63	0.41	0.43	0.52	0.46
Family	0.15	0.31	0.19	0.32	0.13	0.19	0.24	0.28	0.25
Immunology	0.65	0.35	0.44	0.35	0.57	0.38	0.57	0.42	0.46
Psychiatry	0.54	0.38	0.38	0.34	0.65	0.40	0.34	0.65	0.40
**Nephrology**	**0.72**	**0.32**	**0.43**	0.38	0.63	0.33	0.55	0.42	0.46
Geriatrics	0.15	0.34	0.18	0.00	0.65	0.01	0.25	0.28	0.24
Respiratory	0.59	0.32	0.41	0.38	0.6	0.44	0.40	0.57	0.44
Infectious disease	0.01	0.3	0.01	0.01	0.67	0.01	0.05	0.04	0.04

For our specific use-case (i.e., calculating average wait time from family physician referral to first specialist visit), in collaboration with clinical specialists, we determine that precision matters more than recall. More specifically, in-order to be confident with the wait time estimates made from our automated predictions, models need to achieve a precision of at least 0.70 and a recall of at least 0.30. This decision narrows the number of specialties that can be analyzed down to nine. Table 4 presents a more in-depth evaluation of the performance of the best models for the selected specialties.

**Table 4:** Precision, recall, specificity, negative predictive value (NPV), and Brier score loss for the models which we used for prediction. The standard deviations for each measurement are presented between parentheses.

Specialist Type	Precision (Std.)	Recall (Std.)	Specificity (Std.)	NPV (Std.)	Brier Loss (Std.)
General surgery	0.72 (0.09)	0.31 (0.11)	0.98 (0.02)	0.89 (0.01)	0.09 (0.01)
Cardiology	0.83 (0.10)	0.41 (0.05)	1.00 (0.00)	0.98 (0.00)	0.02 (0.00)
OBGYN	0.95 (0.03)	0.49 (0.12)	1.00 (0.00)	0.93 (0.01)	0.05 (0.01)
ENT	0.98 (0.01)	0.42 (0.08)	1.00 (0.00)	0.93 (0.01)	0.05 (0.01)
Dermatology	0.85 (0.06)	0.51 (0.17)	0.98 (0.01)	0.90 (0.03)	0.07 (0.02)
Neurology	0.82 (0.09)	0.33 (0.09)	1.00 (0.00)	0.98 (0.00)	0.02 (0.00)
Urology	0.91 (0.02)	0.52 (0.13)	1.00 (0.00)	0.97 (0.01)	0.02 (0.01)
Rheumatology	0.78 (0.09)	0.38 (0.08)	1.00 (0.00)	0.99 (0.00)	0.01 (0.00)
Nephrology	0.72 (0.10)	0.32 (0.15)	1.00 (0.00)	1.00 (0.00)	0.00 (0.00)

As observed in Table 3, there is a large variation in performance between specialties. To explore the possible causes of this variation, we study the effects of: 1) the number of training examples on performance, and 2) the number of tokens in a note on classification accuracy.

### Effect of number of training examples on performance

**Fig 2:**
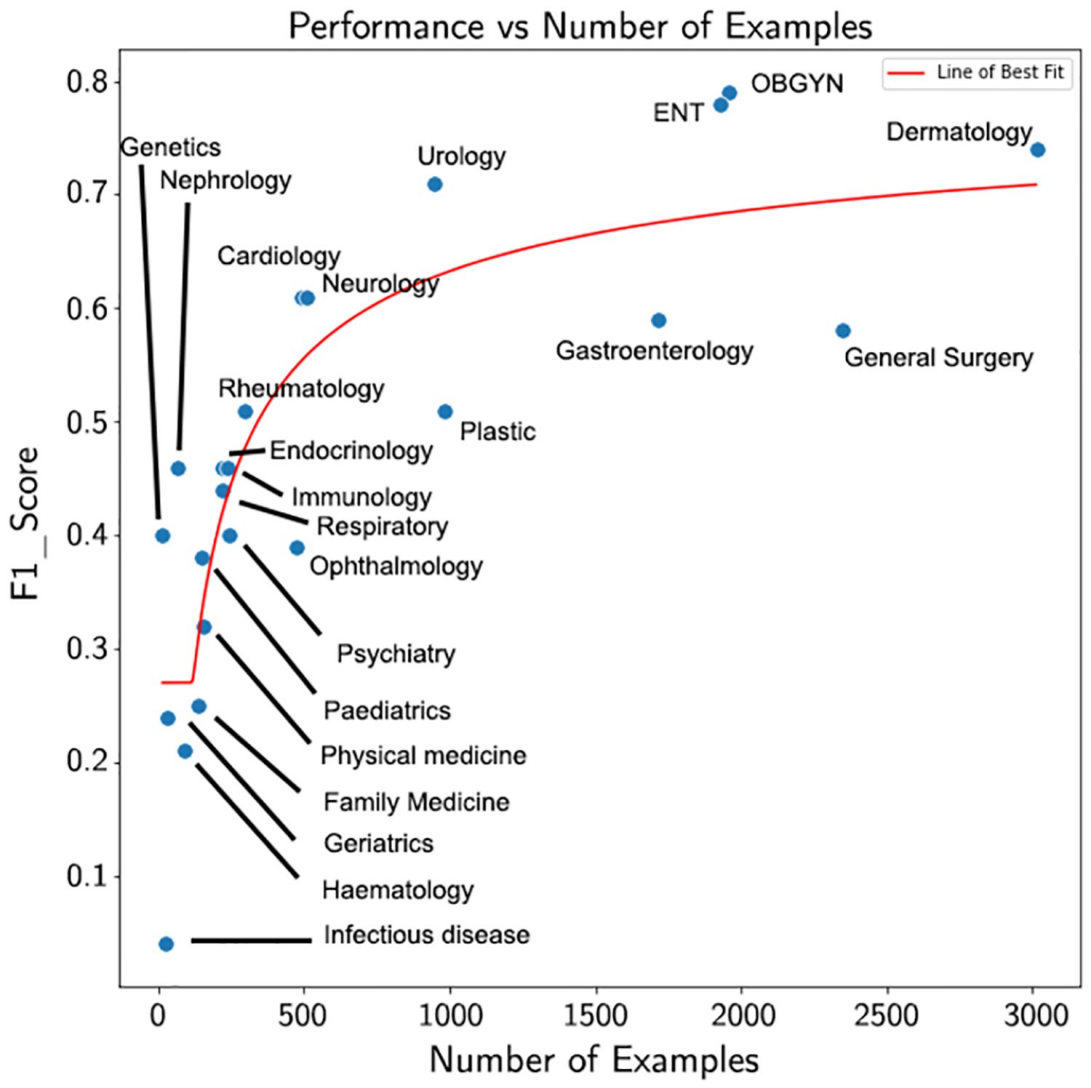
The *F*_1_Score as a function of the number of training examples. In this figure we present the *F*_1_ score from the model with the highest *F*_1_ score for each specialty (the last column of Table 3) against the number of training (Table 1).

First, we explore the relationship between number of training examples and performance. Fig 2 plots the *F*_1_Score on the y-axis and the number of examples on the x-axis. We observe that more training examples generally means better performance. However, for certain specialties (e.g., nephrology), good performance was achieved without many examples. On the other hand, there are specialties with many examples (i.e., notes) that perform poorly (when compared to other specialties with a similar number of notes), such as general surgery.

These observations can likely be explained by examining the uniqueness of a specialty. A specialty that examines the same body parts, and shares procedures and terms relevant to other specialties is likely to be harder to classify than specialties that overlap less with other specialties (e.g., OGBYN).

### Effect of length of note on performance

**Fig 3:**
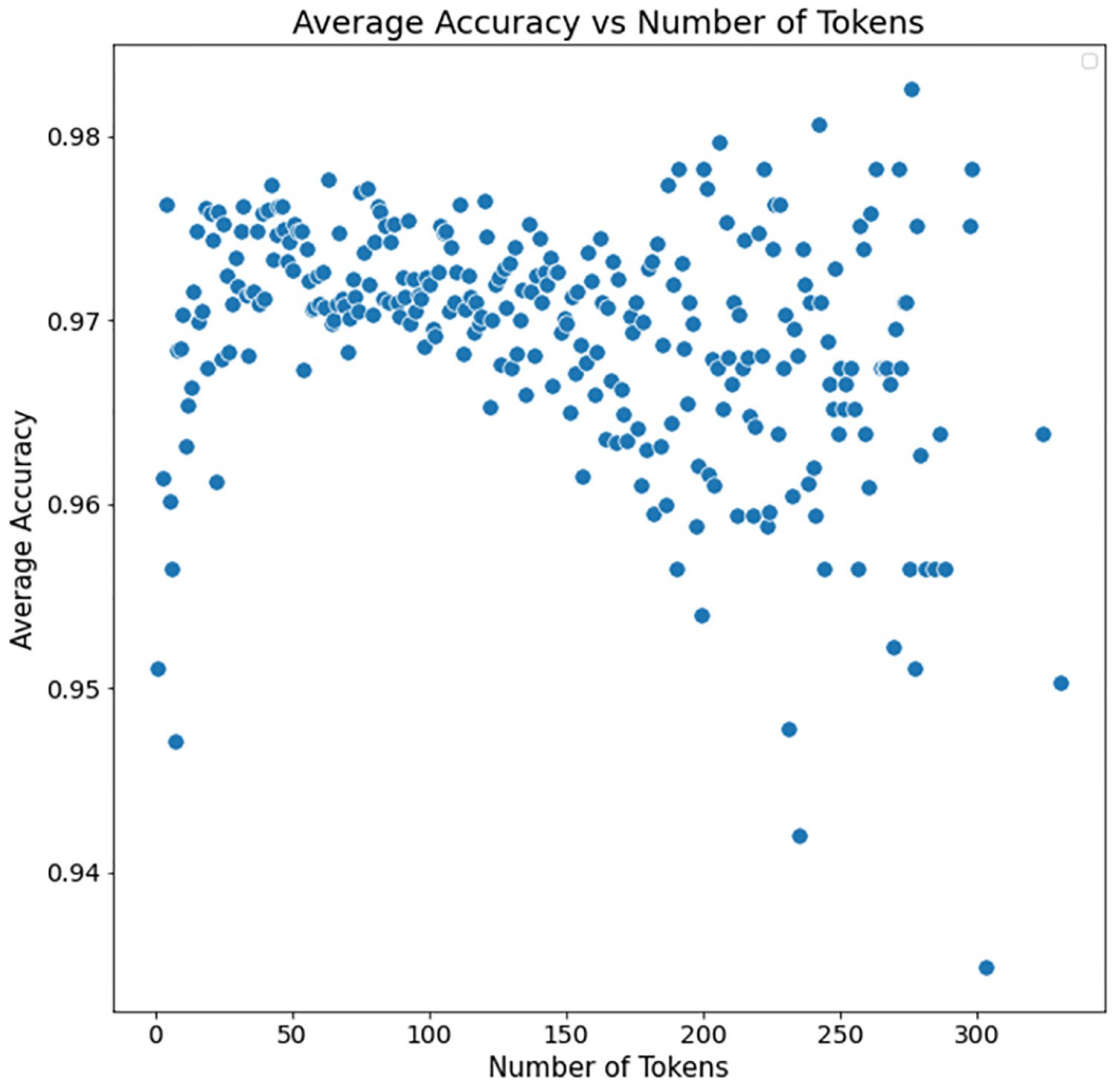
The average accuracy (across all specialties) for the highest performing classifiers (when optimized for *F*_1_Score) by different lengths of the referral notes (as measured by number of tokens). We observe that notes with few tokens have poor accuracy. On the other hand, notes with many tokens are likely have a noisy signal (containing more keywords belonging to other specialties), thus resulting in poor performance.

In this section, we examined the relationships between classification performance (accuracy) and length of referral note (as measured by number of tokens). Fig 3 plots the number of tokens in a referral note against the average accuracy of models on referral notes averaged across all specialties. For the best model of each specialty, we individually calculated the accuracy of the model on all the notes of a particular length (i.e., number of tokens). For example, if there are 5 classifiers and 10 notes of a particular size: we would separately calculate the accuracy of each of those classifiers on the 10 notes (resulting in 10 accuracy values). We then aggregated these accuracy measurements for each number of tokens by averaging the accuracy of all classifiers for each length (i.e., each presented value would be the average of the accuracy of all 5 classifier in the example). Values of “number of tokens” that had fewer than 5 associated notes are not plotted (in accordance with ICES privacy guidelines). See S1 Appendix which provides the average accuracies associated with each note length.

We observe that having very few tokens (e.g., <10) or very many tokens (e.g., >125) results in sub-optimal performance (when compared to notes having between 50 and 100 tokens). We observe, from manual examination of the notes, that very short notes are not likely to be informative. That is, “Please see John Smith” does not allow either human annotators or an automated algorithm to predict the specialist accurately. For this reason, we did not train on or classify any notes with fewer than six tokens (enough to convey “Please see John for chest pain”). Longer notes are more likely to include patient history including diseases and symptoms unrelated to the target specialty which increases the likelihood of misclassification.

### Calculating wait times

We only calculate and present wait time estimates from FP referral to specialist visit for specialists who have a model meeting our determined threshold. That is, if the performance of the best model for a particular specialty does not pass the requirements (precision of at least 0.70 and recall of at least 0.30) then we did not attempt to estimate the wait time. This decision is based on expert feedback of what percentage of notes should be captured and how precise the measurement should be to provide a trustworthy measurement. However, we acknowledge that for different methods or different datasets other minimum thresholds may be more appropriate.

To calculate the wait times, we deploy the best ML algorithm for each specialty on all EMR referral notes from 2015 (N = 174,190), i.e., the full study cohort which includes those with multiple referral notes that were not used in the development and evaluation of the machine learning approach. For each patient, for each specialty, we measure the wait time as the number of days between the first FP referral to a specialist physician and the first consultation visit of the same specialist physician, Fig 1.

To allow for adequate recall of FP referrals made at the end of the calendar year, when measuring the wait times for referrals made in 2015, we allowed for referrals to match to consultation visits occurring in 2016 (e.g., referral in November 2015 and first specialist visit in April 2016).

**Fig 4:**
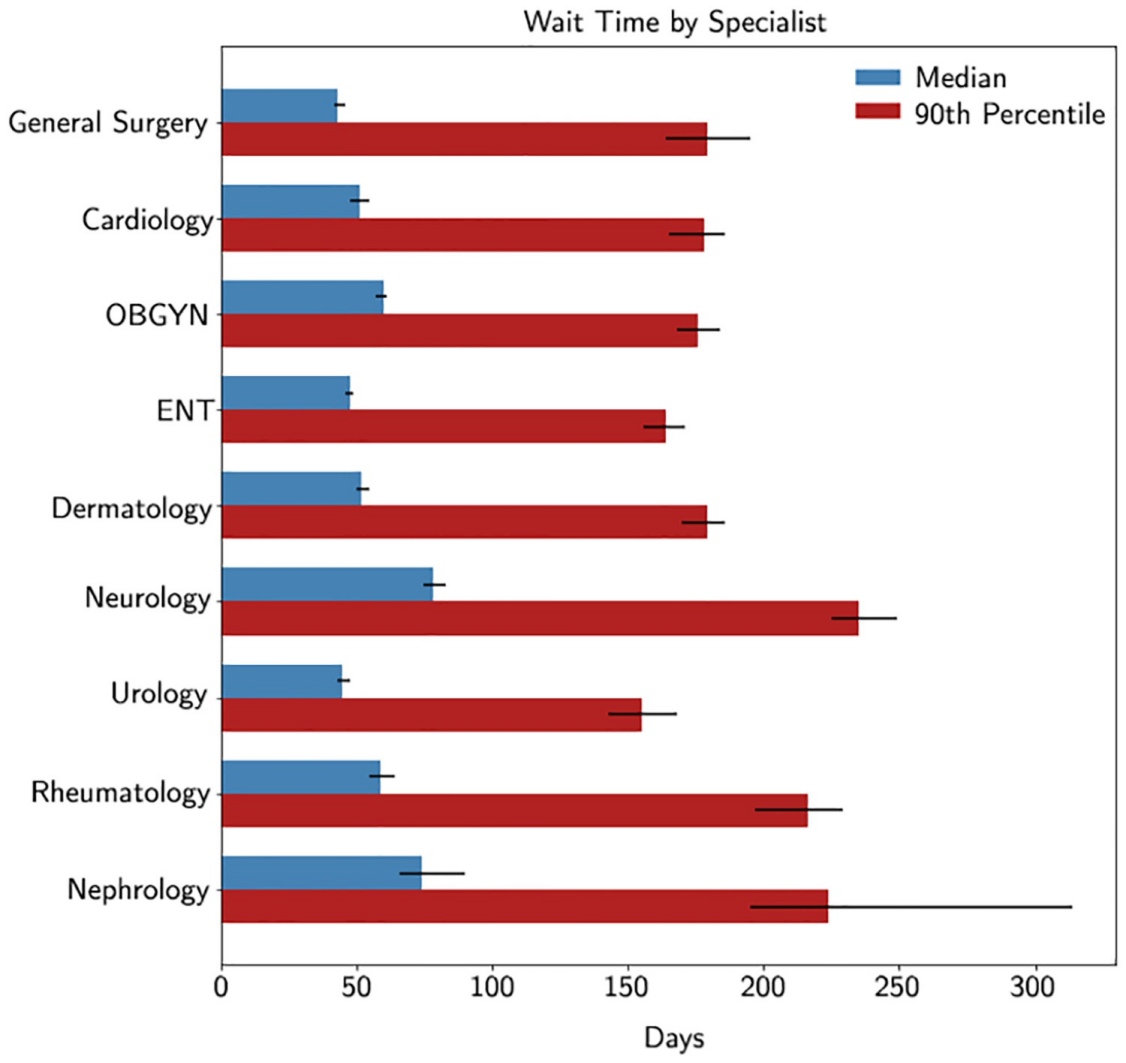
The estimated median and 90^th^ percentile wait time from family physician referral to specialist visit for nine specialties. The error bars represent the 95% confidence interval (arrived at by bootstrapping).

These models are applied on the full set of referral notes from 2015 which had at least 6 tokens (N = 174,190). Fig 4 presents the median and 90^th^ percentile wait times for target specialties matching our evaluation criteria.

**Fig 5:**
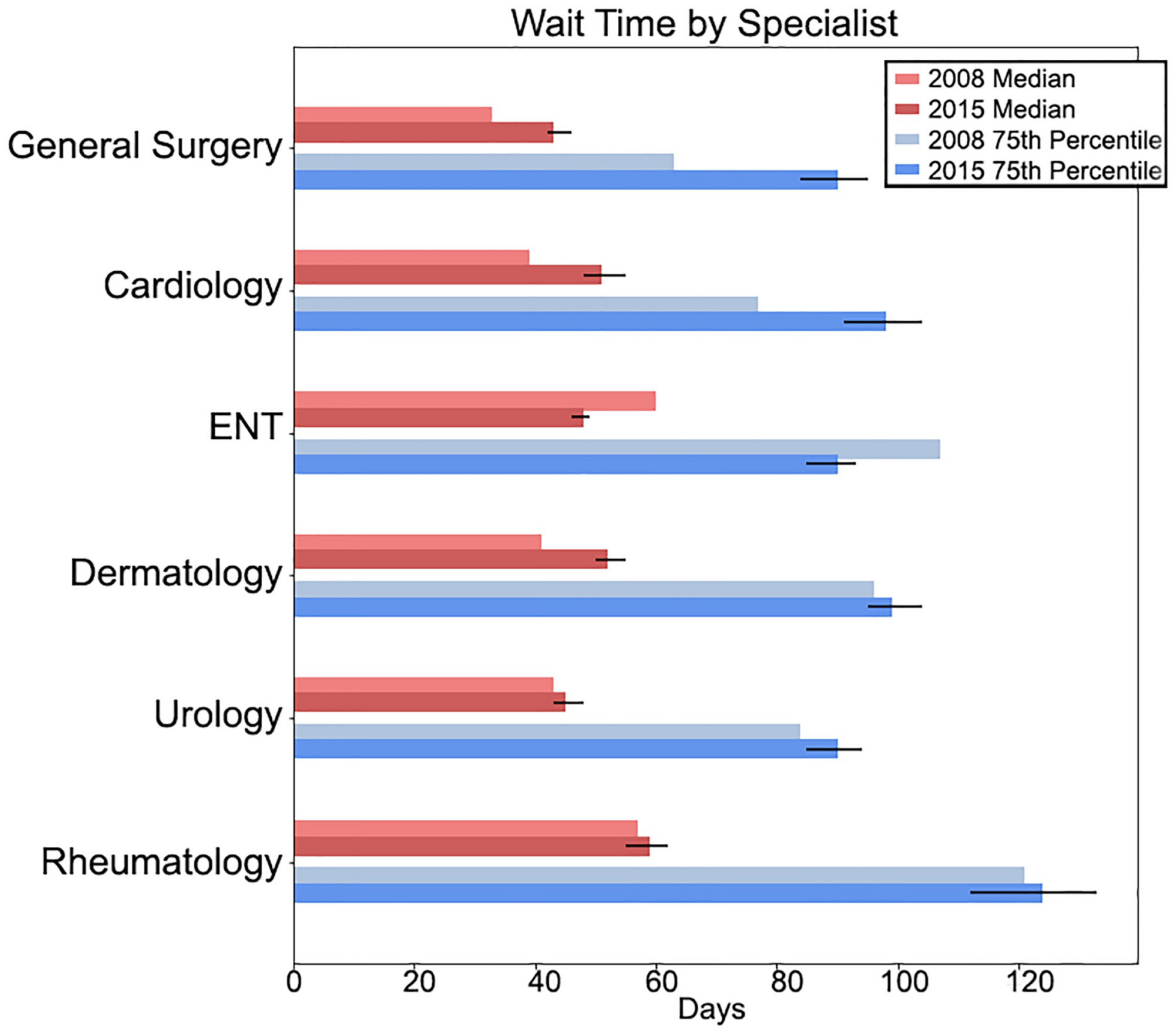
The estimated median and 75^th^ percentile wait time from family physician referral to specialist visit for four specialties. The error bars represent 95% confidence interval (arrived at by bootstrapping). The 2008 numbers are from our previous work and relied on manual coding.

Fig 5 compares the estimated wait times in 2015 against the latest available manually human coded estimates [11]. As the method for labelling referral notes is different the conclusions that can be made from this comparison should be made with caution. However, having developed these automated approaches, future comparisons should not be so costly to perform and would enable timely measurements of the effects of health policy on primary care wait times.

### Performance and estimated wait times

**Fig 6:**
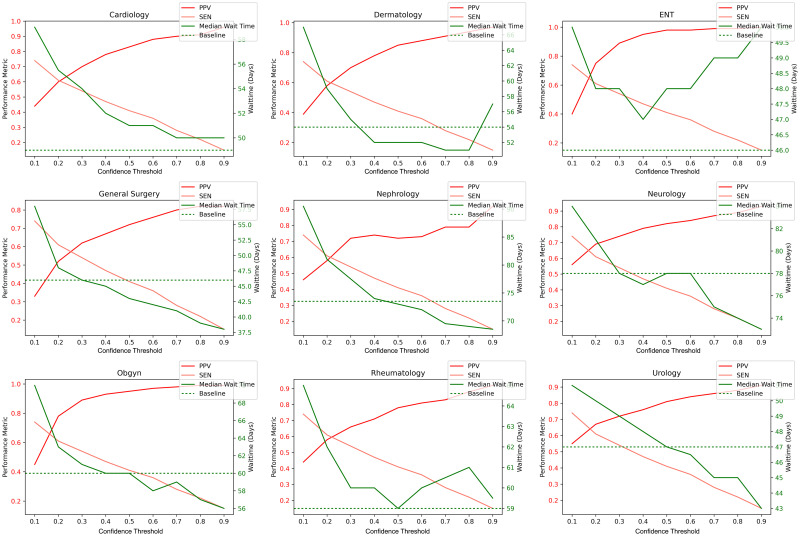
The effect of different confidence thresholds (x-axis) on classification metrics (left y-axis) and estimated median wait times (right y-axis) for the 9 selected specialties. The dashed green line represents the “baseline” wait-time: the wait time if we only estimated using our gold labels (i.e., using patients who only ever had a single referral and specialist consultation in 2015).

In this section, we explore the effect of choosing different confidence thresholds for the predictions of our models. Previously, we used the confidence threshold of 0.5 on classifiers which have a performance of at least 70% PPV and 30% sensitivity. As this system is being developed for a specific application purposes in our healthcare system, the threshold was selected through expert consultation with health-system researchers from our system. However, we now explore the effects of possibly changing the confidence threshold (and thus the performance thresholds), as might be desired by other systems or researchers. For each model represented in Fig 4, we plot how different confidence thresholds affect predictive performance (PPV and sensitivity) and estimated wait-times Fig 6. We observe that the confidence threshold of 0.5 (PPV> = 70% and sensitivity> = 30%) often coincides with a “stabilization” or decrease in slope for estimated wait times (e.g., clear examples can be seen in cardiology and dermatology among others). We observe that the median wait time does not differ substantially when measured either using only notes in our gold-standard set or using machine prediction to capture additional patients. However, depending on the use-case, classification might still be justified to increase the number of patients composing a class (and thus decreasing the 95% confidence interval) to allow for meaningful significance testing between different time periods.

## Discussion

FPs EMRs contain a wealth of information about patients seen in primary care. In Ontario, there are currently 14 EMR vendors used by family physicians [40]. Unfortunately, the data held within these EMRs are not documented in a consistent way. Traditionally, extracting free-text EMR data required abstractors to review charts and code the data. ML provides the opportunity to more efficiently code the free text portion of EMRs. We were able to use ML on the free-text portion of FP EMR’s referral note to classify the target specialist physician for that referral, a task which previously had to be done by humans.

The high precision of ML using unstructured EMR data allows timely results at a lower cost and on a larger scale. From a primary care perspective, this information can help inform decision makers and providers about which patients or FP practices are experiencing long wait times in seeing specialist physicians. This work can also be used by health policy researchers to examine, in a timely manner, the effects of policies.

### Limitations

While using automated algorithms enables classification of more referral notes, the low recall scores (0.30–0.50) make our results susceptible to skews caused by non-random mis-predictions by the algorithm. That is, although our automated method enabled us to label, on average, 167% more notes for each specialty when compared to the previous study of Jaakkimainen *et al*. [11], manual labelling is needed to determine if the captured notes are truly representative of the entire sample.

Furthermore, our training set was relatively small thus limiting the performance we could achieve. Our experiments demonstrate that having more notes often results in increased performance.

Another limitation is the lack of human-curated labels for referral notes. To address this limitation without incurring the high costs of human labelling, we devised a method to accurately infer the labels from administrative data: we extracted labels for patients who had only one referral and one consultation visit in 2015. However, this method limits our training set size and subsequently resulted in us not being able to train classifiers for specialties such as orthopedics where many of the referrals used custom forms thereby reducing our ability to automatically capture such labels. Furthermore, unlike a manually labelled random sample, work is needed to demonstrate that idiosyncrasies of the predictive algorithm don’t change the population being labelled.

The content of referral notes also vary amongst FPs and clinics. Busy FPs may write a referral using personalized abbreviations or few words (fewer tokens) per referral note thus making it more difficult to predict. The use of custom-stamps or forms which used tick-boxes in their referrals may also mask some of the words that could be used to label a referral note. While cross-validation would capture much of these differences on our specific dataset, more work is needed to ensure the generalizability and validity of predictions on larger and external datasets that have not been labelled (e.g., notes from later years or notes from FPs which are not in our training set).

We find an increase in the median and 90^th^ percentile of wait times for most specialties when compared to 2008 data. However, a direct comparison cannot be made due to the differences in referral note labelling (i.e., automated vs. human labelling). While a direct comparison cannot be made, the result from such a comparison matches those from Canadian-based surveys similarly found increased wait times in seeing specialist physicians [4, 41].

## Conclusions

In this paper, we used ML to predict the target specialist physician of referral notes to enable the estimation of wait times by specialty. This is significant as, previously, this task needed human abstractors to label the notes. This work represents a reduction in the cost of gathering such metrics and an increase in the speed and frequency with which health system researchers can gather such metrics. Furthermore, using ML in this way to clean untrustworthy or incomplete labels can be employed for other applications in health system research. We found that of the 6 specialties for which wait times were estimated using both 2008 and 2015 data, 2 had a substantial increase in both median and 75th percentile wait times, one had a substantial decrease in both median and 75th percentile wait times, and 3 has non-substantial increases. In future work, we will gather and explore a larger time-series of referrals to enable direct comparison between different dates, thus enabling the evaluation of quality improvement initiatives.

## Supporting information

S1 Appendix Table of the average accuracy (across all specialties) for the highest performing classifiers (when optimized for *F*_1_Score) by different lengths of the referral notes (as measured by number of tokens). Visually represented in Fig 3.
(XLSX)

## Abbreviations

CPP: cumulative patient profile
EMRs: electronic medical records
ENT: Otolaryngology
FP: family physician
ML: machine learning
OBGYN: Obstetrics and Gynecology
OHIP: Ontario health insurance plan
PPV: positive predictive value
tf-idf: term frequency inverse document frequency
